# Hollow Dodecahedra Graphene Oxide- Cuprous Oxide Nanocomposites With Effective Photocatalytic and Bactericidal Activity

**DOI:** 10.3389/fchem.2021.755836

**Published:** 2021-09-09

**Authors:** Zezhi Shan, Yanrong Yang, Haoran Shi, Jiali Zhu, Xiao Tan, Yi Luan, Zhenqi Jiang, Ping Wang, Jieling Qin

**Affiliations:** ^1^ Tongji University Cancer Center, Shanghai Tenth People's Hospital, School of Medicine, Tongji University, Shanghai, China; ^2^ Department of Colorectal Surgery, Fudan University Shanghai Cancer Center, Department of Oncology, Shanghai Medical College, Fudan University, Shanghai, China; ^3^ School of Life Sciences, Shandong University of Technology, Zibo, China; ^4^ Institute of Engineering Medicine, Beijing Institute of Technology, Beijing, China

**Keywords:** Cu_2_O, dodecahedra, graphene oxide, photocatalytic performance, antibacterial effect

## Abstract

In this study, a kind of graphene oxide-cuprous oxide (GO-Cu_2_O) nanocomposites was fabricated with different morphologies to serve as a photocatalytic material for the degradation of organic/inorganic dyes under visible light and the bactericidal effect against pathogenic bacteria. The GO-Cu_2_O was prepared with solid cube and hollow dodecahedra morphologies through *in-situ* synthesis, and characterized by scanning electron microscopy (SEM), transmission electron microscope (TEM), X-ray diffraction (XRD), Raman, Ultraviolet and visible spectrophotometry (UV/vis), and Fourier transform infrared spectroscopy. In comparison with cubic GO-Cu_2_O, the absorption and degradation efficiency of the GO-Cu_2_O dodecahedra (GCD) composite in Methyl orange (MO), Rhodamine B (RhB), and phenol was higher owning to the more active sites for the simultaneous dye and light absorption of hollow structure. The antibacterial effect of the GO-Cu2O dodecahedra was examined by the flat colony counting method with an excellent bactericidal effect against pathogenic bacteria. The possible mechanism for the preparation of GCD possessing the enhancement of the visible-light photocatalytic and antibacterial efficiencies were also investigated.

## Introduction

Water pollution causes great damage to ecosystems, human health, as well as the sustainable economic and social development because the pollutant complex, along with bacteria, cause difficulty in decontamination by conventional water treatment processes (Schwarzenbach et al., 2010; Wang and Yang, 2016). Hence, developing an effective and facile way to degrade pollutants has become an active area in environmental research. Recently, inorganic nanomaterials have attracted numerous attentions because of their controllable shapes and sizes, as well as their effective photocatalytic activities, such as those in metal oxide semiconductors (e.g. TiO_2_, ZnO) or narrow band gap semiconductors (e.g. Ag_3_PO_4_) (Shao et al., 2019; Zhang et al., 2020).

Although these inorganic materials exhibit promising photocatalytic activities, there are still several problems that need to be overcome, for instance, relatively poor light-harvesting abilities in the visible region, the use of toxic or harmful chemicals, or poor charge separation and transport. Cuprous oxide (Cu_2_O) is a promising metal oxide material in the application of photocatalysis because it is a p-type semiconductor (C _hole_ > C _electron_) with a small band gap (E_g_ = 2.17 eV). Recently, numerous efforts have been devoted to synthesize Cu_2_O with different morphologies such as nanowires, octahedra, cuboctahedra, and nanocubes (Hua et al., 2011; Deng et al., 2012; Hou et al., 2013; Hong et al., 2014). Among them, a hollow structure has intrinsic advantages in photocatalysis applications Xiao et al. (2019), such as enhancing light harvesting Li et al. (2007), Wang et al. (2012), Wu et al. (2013), Dinh et al. (2014), Qi et al. (2014), promoting the synergistic effects of light scattering and localized surface plasmon resonance (LSPR) Zhang et al. (2014), Shi et al. (2016), reducing charge recombination Marschall (2014), Li et al. (2015), and accelerating surface reactions due to a high surface area (Sun et al., 2003; Vaughn and Schaak, 2012; Wang et al., 2017). Furthermore, due to a large carbon sheet structure, graphene oxide (GO) was also introduced in a hybrid with Cu_2_O to effectively increase the adsorption sites and improve the transfer of electrons between the materials for the inhibition of hole and electron recombination. The addition of carbon based 2D materials during the *in-situ* preparation of the nanoparticles may improve the photocatalytic performance effectively (Khan et al., 2015; Khan et al., 2016a; Khan et al., 2016b; Khan et al., 2017; Ahmed and Haider, 2018; Khan et al., 2018). Lee and his group synthesized Ag-Cu_2_O together with graphene oxide for the enhanced photocatalytic performance (Sharma et al., 2018). SunilMeti et al. reported zinc oxide nanocomposites wrapped with reduced graphene oxide to enhance the photocatalytic activity (Meti et al., 2018).

**SCHEME 1:**
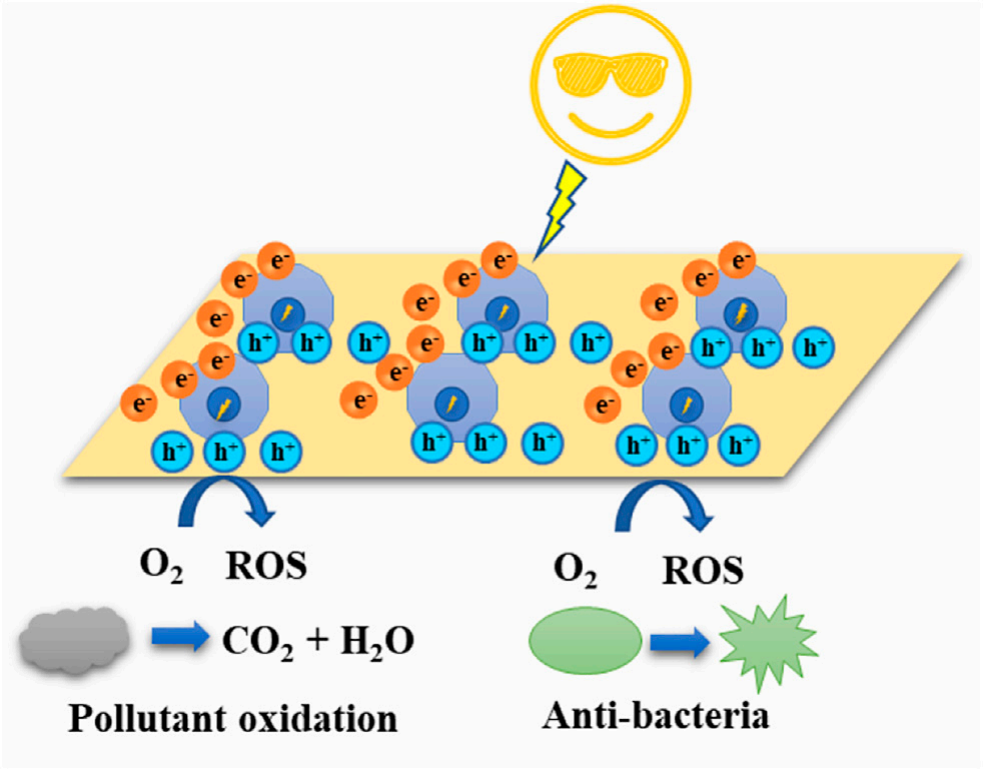
Possible photocatalytic and bactericidal mechanism of GCD.

Herein, the nontoxic and novel visible-light-driven GO-Cu_2_O composite was used as an inspiring photocatalytic material to address the aforementioned problems in water pollution. In this work, aqueous solutions of copper salt, alkali, surfactant, and reductant were used to prepare hollow dodecahedral Cu_2_O through an *in-situ* synthesis process. The GO sheet was added through an electrostatic reaction of negatively charged GO and Cu ions and leaving a final modified GO-Cu_2_O hollow dodecahedral (GCD) structure. The solid cubic GO-Cu_2_O structures were also fabricated by systematically changing the reductant amount for comparison. After the successful preparation of the crystal components, the structures were characterized and confirmed. The photocatalytic performance of the GO-Cu_2_O in different dyes under visible light were investigated and compared, while the antibacterial performance was evaluated by a flat colony counting method and TEM. In general, the as-prepared GCD enhanced light-harvesting, separated the excited e^−^-h^+^ pairs, and promoted charge transfer. The increased reactive oxygen species generated from visible irradiation made the oxidation of the organic pollutant and elimination of the bacteria possible, as shown in Scheme 1.

## Experimental Details

### Materials and Characterization

Anhydrous copper (II) chloride (CuCl_2_; 97%), hydroxylamine hydrochloride (NH_2_OHHCl; 99%), sodium hydroxide (98.2%), sodium dodecyl sulfate (SDS; 100%), graphite and methyl alcohol are purchased from Sinopharm Chemical Reagent limited corporation. Graphene oxide (GO) is synthesized from graphite using the improved Hummers method with additional KMnO_4_ (Yang et al., 2011; Yang et al., 2013a; Yang et al., 2013b; Qin et al., 2015). All chemicals are used as obtained without further purification. Deionized water is used in all the procedures.

The characterizations, photocatalytic activity, and bactericidal activity tests were described in the supporting information.

### Synthesis of Graphene Oxide-Cuprous Oxide Nanocomposites With Different Morphologies

In a typical synthesis, different volumes of water were used to obtain the final 1 L solution (Deng et al., 2012; Huang et al., 2012). Flasks containing CuCl_2_ solution (0.1 M, 50 ml) and sodium dodecyl sulfate (8.7 g, SDS; 100%) were placed in a water bath and kept at 32°C. Then, NaOH (1.0 M, 18 ml) was added in dropwise with vigorous stirring. A Cu(OH)_2_ precipitate was formed and the color of the solution changed from dark blue to light blue. Different amounts of NH_2_OHHCl (solid cube: 40 ml; hollow dodecahedra: 240 ml) were then poured within 5 s and left to cool down to RT. The final products were then dried and obtained after washing several times with DI water/ethanol. The GCD nanocomposites were synthesized via a similar route as the Cu_2_O, except that 40 mg GO was sonicated into the deionized water at the beginning.

## Results and Discussion

With the addition of the NH_2_OHHCl reductant during the *in-situ* process, the Cu(OH)_2_ was the first nucleation seed and then the Cu_2_O nanocrystals grew on the surface to form various morphologies. The higher concentration of the NH_2_OHHCl increased the growth rate of the Cu_2_O and changed the morphology from a cube to a dodecahedron, while the HCl from the reductant etched the Cu_2_O to form the final hollow structure (Kuo and Huang, 2008).

**FIGURE 1:**
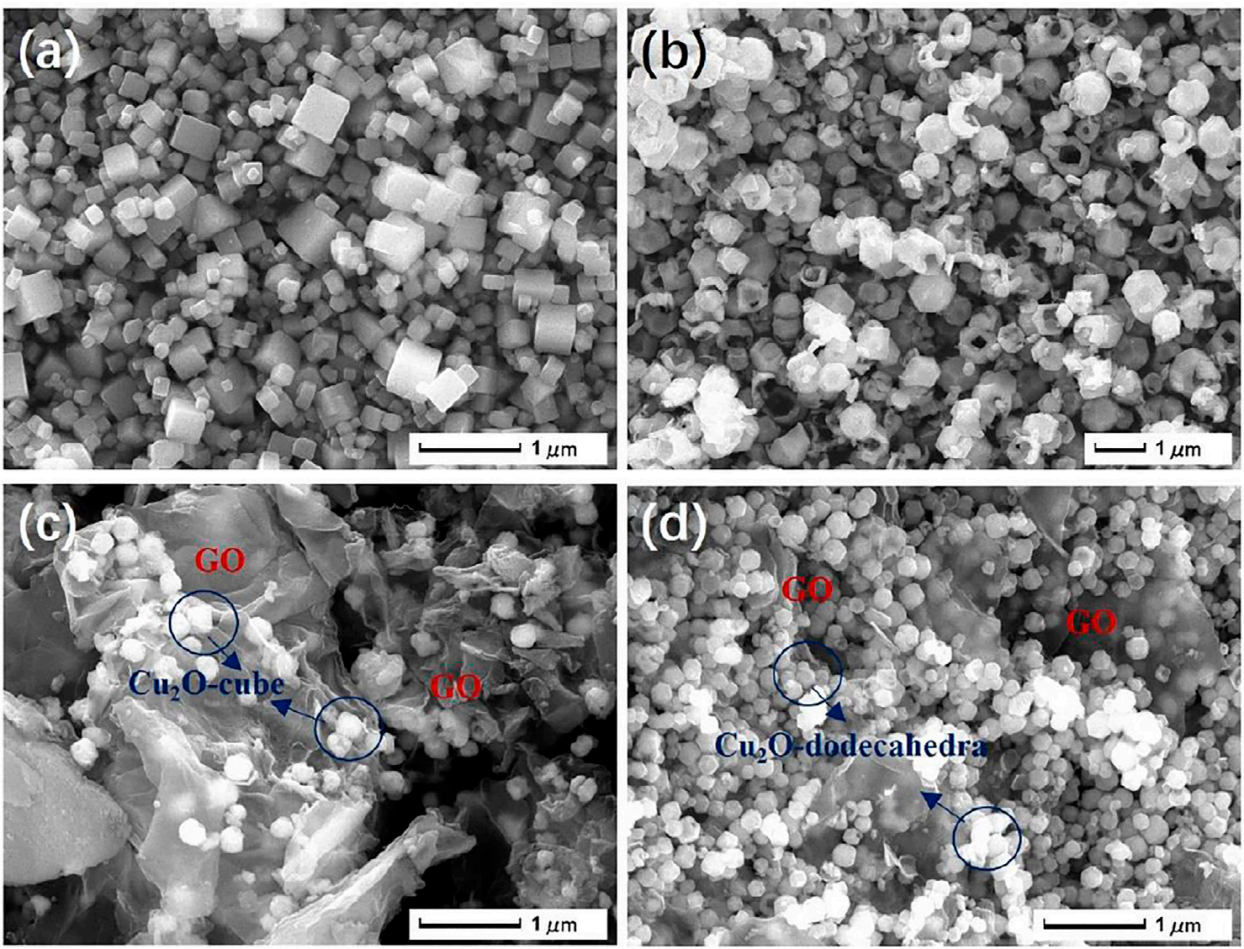
The SEM images of CC **(A)**, CD **(B)**, GCC **(C)**, and the GCD nanocomposite **(D)**.

The morphologies of the samples were characterized by SEM. Figure 1A showed that the synthesized CC possessed a cube morphology of about 100–500 nm in size. From the SEM image in Figure 1B, CD displayed a dodecahedron morphology, and the average diameter was about 300 nm, most of which were broken or full of holes. After the addition of GO to the samples, the ionic interaction between the Cu cations and GO^−^ anions modified the Cu_2_O morphology. The SEM image of the GCC composites (Figure 1C) highlighted the presence of cube-like Cu_2_O polyhedrons with a uniform size of less than 200 nm, most of which were wrapped by GO sheets. The addition of GO also initiated a minor change of the morphology. The GCD in Figure 1D illustrated that the dodecahedron-like particles have an average diameter of about 100–200 nm.

**FIGURE 2:**
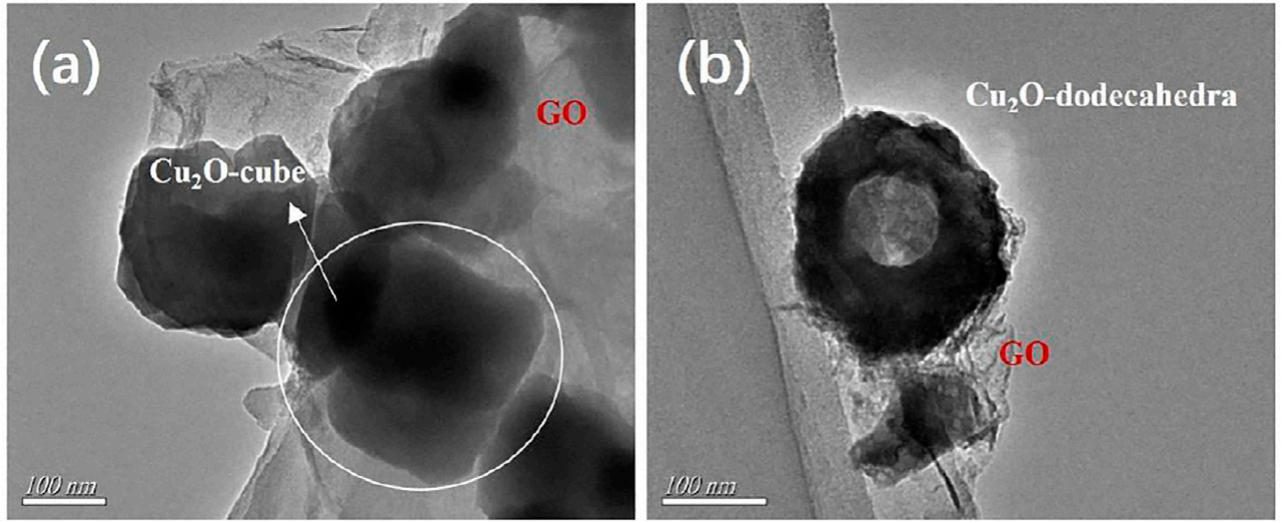
**(A–B)** TEM images of the GCC nanocomposite **(A)** and the GCD nanocomposite **(B)**.

The solid cubes and hollow dodecahedra were also characterized by TEM. The size of the CC particles coated with the GO sheets (Figure 2A) was estimated to be between 150–200 nm, which was consistent with the SEM image of the GCC particles in Figure 1C. Figure 2B was a typical TEM image of CD covered with a small quantity of GO sheets, from which the hollow structure and the presence of GO were clear.

**FIGURE 3:**
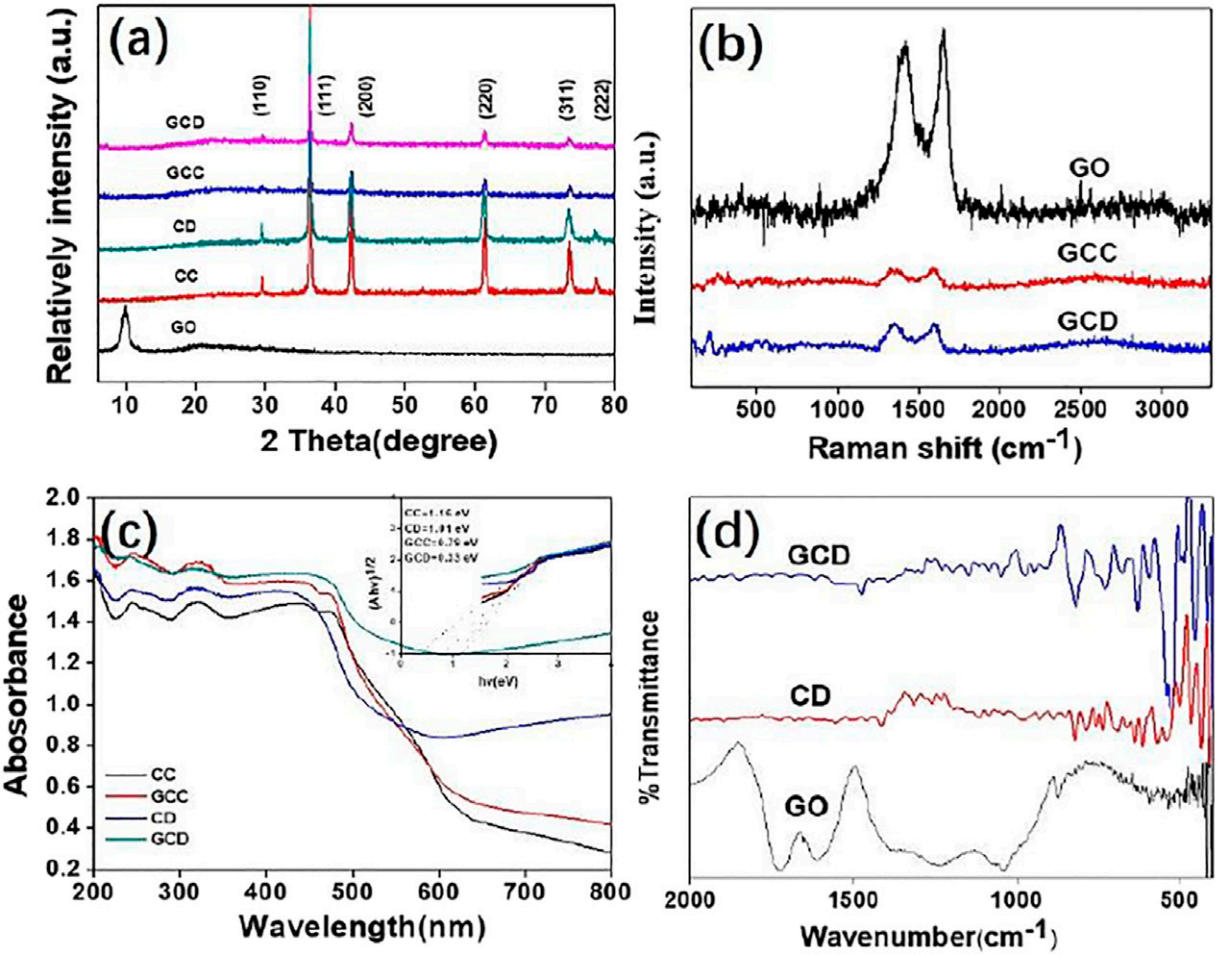
XRD patterns of GO and GO-Cu2O with different morphologies **(A)**; Raman spectra of GO and GO-Cu2O with different morphologies **(B)**; UV/vis and the plot of light energy (αhν)1/2 vs energy (hν) **(C)**; FT-IR spectra of GO, Cu2O, and GO-Cu2O **(D)**.

After determining the composites’ morphologies by SEM and TEM, the XRD spectra were utilized to confirm the prepared crystal components. According to the JCPDs, the peaks at (110), (111), (200), (220), (311), and (222) were assigned to the standard cubic structure Cu_2_O (No. 03–0898) (Wang et al., 2002; Zhang et al., 2007). No other peaks were detected, such as CuO and Cu cupric oxide, demonstrating the purity of the as-obtained products. There was no clear peak for GO observed in the XRD pattern, due to the small amount and low diffraction intensity. The presence of GO was confirmed by Raman and TEM. The (200) diffraction peak of the Cu_2_O cube was stronger than the other diffraction peaks, indicating a high proportion of (100) facets. Similar results were also confirmed for the (220) diffraction peak of the rhombic dodecahedral Cu_2_O nanocrystals, implying a high proportion of (110) crystal planes. Also, Raman spectroscopy was used to measure the vibrations of the sp^2^-hybridized carbon atoms for the confirmation of the presence of GO. Hence, it was observed from Figure 3B that the Raman spectra of the as-synthesized samples contained the D peak (1,343cm^−1^, disorder-activated Raman mode) and G peak (1,588 cm^−1^, sp^2^ hybridized carbon) which were assigned to GO (Yang et al., 2015; Yang et al., 2009; Krishnamoorthy et al., 2012). Figure 3C shows the UV/vis spectra of the as-synthesized CC, CD, GCC, and GCD nanocrystals. Interestingly, after the introduction of GO, the absorption abilities of the GCC and GCD both increased because of the scattering effect of the GO. The inset figure was the Kubelka-Munk transformation of light energy versus energy to calculate the band gap (Yang et al., 2013a; Yang et al., 2013c; Sasca and Popa, 2013). As shown in the figure, the band gaps of the CC, CD, GCC, and GCD were approximately 1.16, 1.01, 0.79, and 0.33 eV respectively. Because of the enhanced light harvesting, slow photons, and the synergistic effect of light-harvesting and LSPR (Xiao et al., 2019), the band gaps of the hollow structures were narrower than the solid structures. After the addition of GO, the proposed band gap of the GO-Cu_2_O composite shifted to lower energies compared with pure Cu_2_O. This indicated that the addition of the GO generated an extra band that served to narrow the band gap of the Cu_2_O for further enhancement of the photocatalytic and bactericidal properties (Yang et al., 2015). In the FT-IR (Figure 3D), the peaks at 1,625 and 1727 cm^−1^ corresponded to the bending and stretching vibrations of O-H and C=O, in the COOH groups of GO sheets, respectively. Also, the C-O and -CH_3_ stretching peaks, and the stretching vibrations of the quinoid ring/benzenoid ring from poly(*o*-anisidine) were around 1,250, 1,386, 1,559, and 1,506 cm^−1^, respectively. Also, the Cu-O stretching vibration of the Cu_2_O was found at 624 cm^−1^. In general, the characterizations from XRD, Raman, UV/vis, and FT-IR spectroscopies confirmed the successful preparation of the nanocomposites.

**FIGURE 4:**
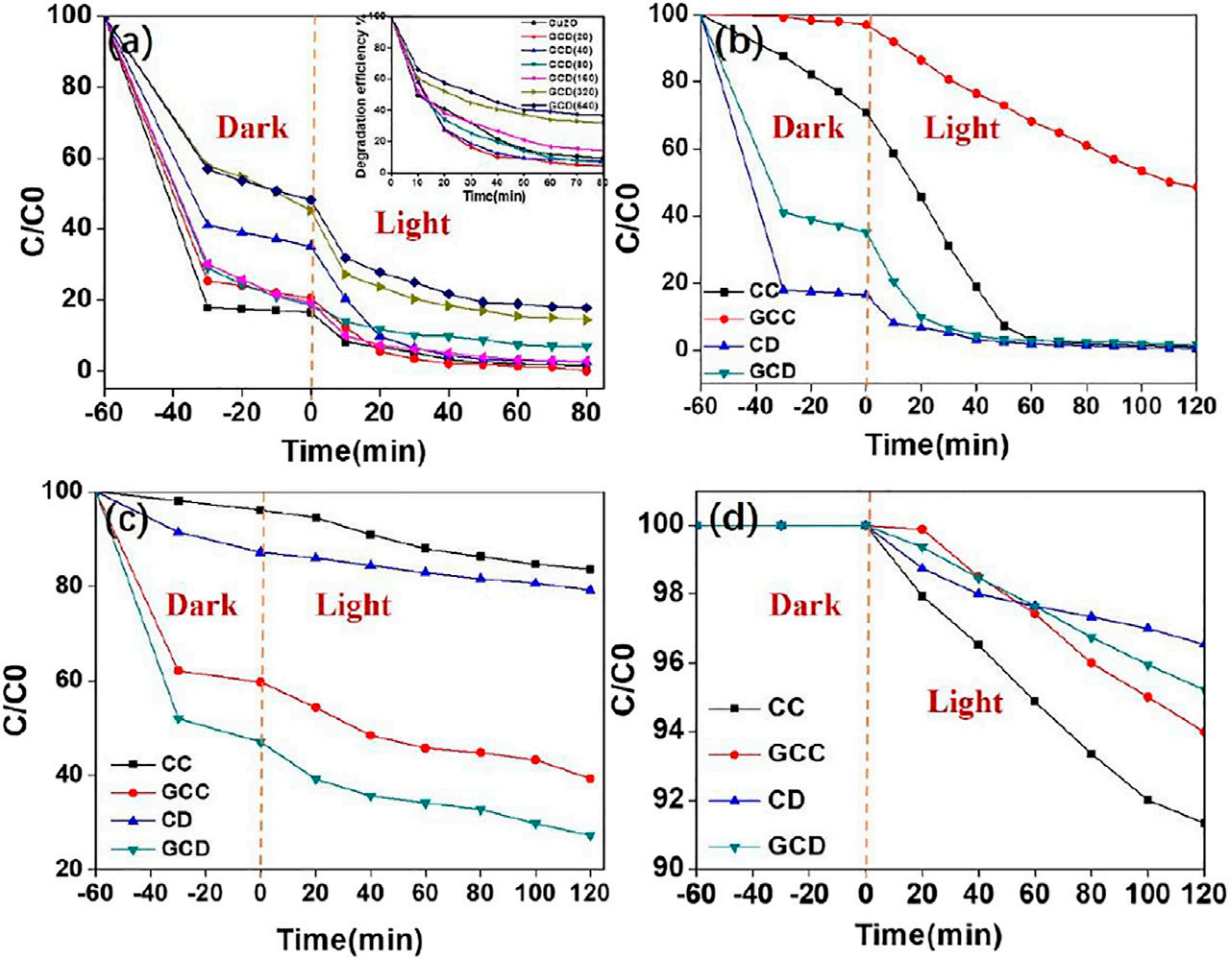
Photocatalytic activity **(A)** and degradation efficiency (inset) of GCD with different amounts of GO for MO degradation under visible light irradiation; different morphologies of GC composites for MO degradation **(B)**; for RhB degradation **(C)**; and for phenol degradation **(D)**.

The photocatalytic activities of the GO-Cu_2_O (50 mg) for the absorption and degradation of various organic pollutants were determined when different photocatalytic dyes (MO-10ppm, RhB-10ppm, Phenol-60ppm) were chosen to react under visible light (Figure 4 and Supplementary Figure S1). Figure 4 1) and (b) demonstrate the degradation curves for methyl orange (MO) using different materials under visible light irradiation. Figure 4A displays the changes in degradation for MO using GCD. It was observed from Figure 4A that after the ultrasonic mixing of the solution for 30 min, the solution was absorbed to some degree. The absorption capacity of pure CD was about 80%, which was higher than the GCD composites. The inset in Figure 4A indicated the degradation efficiency of the GCD increased as the amount of GO. When 40 mg of GO was introduced into the pure Cu_2_O, the corresponding composite showed the highest photocatalytic activities of near 100% within 60 min. However, further addition of GO led to a decrease in the photocatalytic activity of the composites. The possible reason was that too much GO may fully wrap the GCD to eradicate the light irradiation.

To further understand the photocatalytic process, the visible light photocatalytic experiments shown in Figures 4B–D were investigated using MO, RhB, and phenol as target dyes. As shown in Figure 4B, the curve of MO using the GCD was the lowest, whereas the best performance for degrading MO and phenol (Figures 4B, D) was the pure Cu_2_O cube. The efficiencies of absorption and degradation of MO, RhB, and phenol (Figures 4B–D) using the GCD composite were approximately 100%, 75%, and 5% respectively, within 120 min. While the efficiencies of MO, RhB, and phenol using GCC were 50%, 60%, and 6%. Hence, it was suggested that the GCD composite exhibited excellent photocatalytic performance for colored organic dyes. However, the performance of colorless dyes was very poor. This demonstrated that the photocatalytic effect of the GO-Cu_2_O nanoparticles was mainly due to the adsorption of the colored dyes instead of degradation of organic dyes, which was the limitation of the synthesized photocatalytic materials.

Using the experimental results, a possible photocatalytic mechanism was deduced. Generally, the mechanism of the GO-Cu_2_O catalytic activity was similar to other photocatalytic materials. The mechanism consisted of the absorption and degradation of the dyes and the absorption was greater than the degradation in the photocatalytic performance. The possible photocatalytic absorption mechanism was in large part due to the structure of the Cu_2_O. Due to the advantage of a hollow structure, the GCD nanocomposites had the highest absorption. In addition, GO sheets offered more active adsorption sites, which also improved the adsorption of dyes. On the other hand, the photocatalytic reaction was initiated under the irradiation of visible light, leading to the separation of electron-hole pairs in the Cu_2_O. The separated electrons were then excited and moved from the VB to the CB, leaving the holes in the VB. The reactive holes at VB and the reactive oxygen species (ROS) generated through reaction of O_2_ and H_2_O can degrade the dyes. Moreover, GO transferred electrons from the Cu_2_O to the GO sheets which maintained the stability of the material. However, too much GO entangled the Cu_2_O, especially the dodecahedral Cu_2_O, and prevented it from absorbing the colored dyes.

**FIGURE 5:**
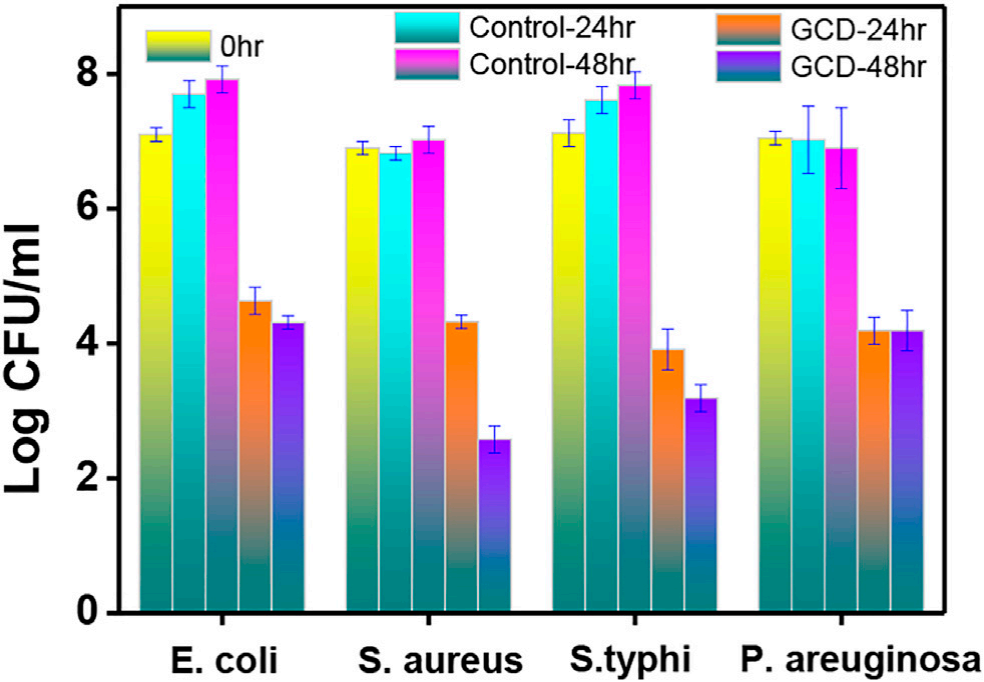
The reduction of the bacteria before/after the addition of GCD.

After the investigation of the photocatalytic performance of GCD, the flat colony counting method was also introduced to examine the specific bactericidal effect (Dahle et al., 2004; Yang et al., 2015; Choudhry, 2016). As shown in Figure 5, the bacterial number was calculated after the addition of GCD at 0, 24, and 48 h. Specifically, the original concentration of the pathogenic bacteria was approximately 1*10^7^ CFU/ml. After the incubation of the bacteria for a period of 24 and 48 h without the GCD, an obvious increase was observed in the *E. coli* and *S. typhi* at 1*10^7.5^ CFU/ml. The amount of *S. aureous* and *P. aeruginosa* remained the same. However, with the treatment of GCD at 24 h, the number of bacteria decreased from 1*10^7.5^ to 1*10^4.5^ CFU/ml with an antibacterial rate of more than 99.9%. After the incubation time was prolonged to 48 h, the number of the *S. aureous* was reduced to 1*10^2.5^ CFU/ml, and the specific bactericidal rate for all the bacteria was summarized in Table 1. Considering the bactericidal experiment, it was concluded that the prepared GCD had a broad-spectrum antibacterial activity toward pathogenic bacteria.

**TABLE 1:** The bactericidal effect of the GCD against different kinds of pathogenic bacteria.

Bacteria	E. coli	S. aureus	S. typhi	P. aeruginosa
Bactericidal effect (%)	99.99	99.999	99.999	99.9

**FIGURE 6:**
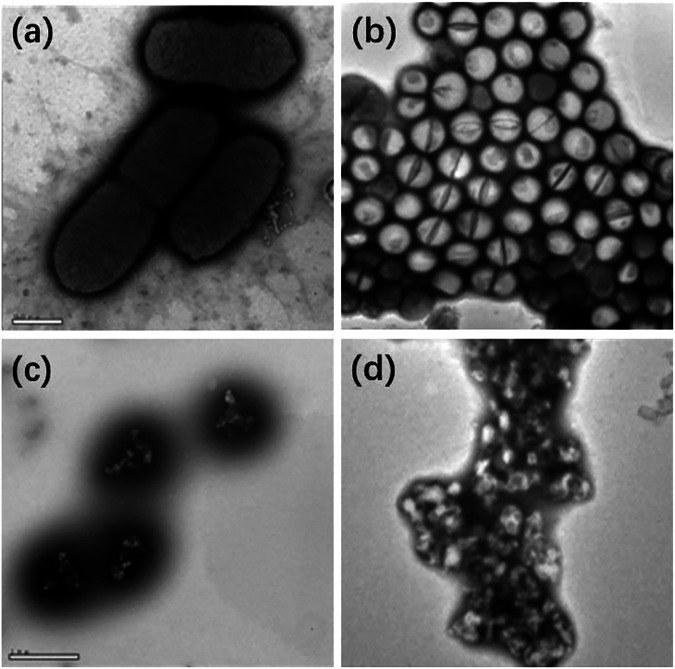
TEM images of E. coli and s. aureus before **(A,B)** and after **(C,D)** the treatment with GCD.

To discuss the mechanism of the bactericidal effect, the morphological changes of *E. coli* and *S. aureus* were investigated by TEM before/after the addition of GCD. Figure 6 (a,b) showed the original *E. coli* rod-like structures with a size of 1 μm × 0.5 μm, while the *S. aureus* has a spherical structure with a diameter of 0.5 μm in the absence of GCD. However, after the treatment of samples that involved the light irradiation of the GCD, ROS was generated to interact with the membrane, making the cytoplasm flow out of the bacteria, and finally killed them. Also, other research considered that the presence of Cu ions in the GCD reacted with the oxygen to produce ROS through the Fenton reaction for further photocatalytic and bactericidal performance (Touati, 2000; El Saeed et al., 2016; Shen et al., 2020). Generally, GCD acted as a kind of bifunctional material for applications in photocatalysis and bactericide.

## Conclusion

In summary, an effective *in-situ* synthesis to produce different morphologies of GO-Cu_2_O was demonstrated. After characterizing with SEM, TEM, XRD, Raman spectroscopy, UV/vis spectroscopy, and FT-IR spectroscopy, the degradation performance of Cu_2_O and GO-Cu_2_O for different dyes under visible light was measured. Antibacterial experiments were also investigated against pathogenic bacteria. The presence of GO along with the hollow structure created a synergistic effect that increased the photo harvesting and facilitated the electron transfer to generate more ROS for the enhancement of the photocatalytic and bactericidal performances. Herein, this work offers new insights into the facile synthesis of GO-based nanocomposites for the applications of photocatalytic degradation and sterilization of wastewater pollutants using visible light.

## Data Availability

The original contributions presented in the study are included in the article/Supplementary Material, further inquiries can be directed to the corresponding author.
