# Supplementary material for: Hollow Dodecahedra Graphene Oxide- Cuprous Oxide Nanocomposites With Effective Photocatalytic and Bactericidal Activity
Source: Front Chem. 2021 Sep 9;9:755836. doi: 10.3389/fchem.2021.755836 (PMC8458578; doi:10.3389/fchem.2021.755836)
Supplement: Supplementary file 1 [file DataSheet1.docx]

**Hollow Dodecahedra Graphene oxide- Cuprous oxide Nanocomposites with Effective Photocatalytic and Bactericidal Activity**

Zezhi Shan^2, #^, Yanrong Yang ^1, #^, Haoran Shi^3^, Jiali Zhu^1^, Xiao Tan ^1^, Yi Luan ^1^, Zhenqi Jiang ^4, ^[[1]](#footnote-1)^*^, Ping Wang ^1, ^[[2]](#footnote-2)^*^, Jieling Qin^1, ^[[3]](#footnote-3)^*^

^1^ Tongji University Cancer Center, Shanghai Tenth People's Hospital, School of Medicine, School of Life Sciences and Technology, Tongji University, Shangshai 200092, China

^2^ Department of Colorectal Surgery, Fudan University Shanghai Cancer Center; Department of Oncology, Shanghai Medical College; Fudan University, Shanghai 200032, China

^3^ School of Life Sciences, Shandong University of Technology, Zibo 255049, China

^4^ Institute of Engineering Medicine, Beijing Institute of Technology, Beijing 100081, China

* Correspondence: [7520200073@bit.edu.cn](mailto:7520200073@bit.edu.cn) (Zhenqi Jiang), pwang@sdut.edu.cn (Ping Wang) and qinjieling770@ hotmail.com (Jieling Qin)

# These authors contributed equally.

**1. Characterization**

Different morphologies of the as-synthesized Cu_2_O samples are examined by field-emission scanning electron microscopy (FESEM, JEOL, JSM-7001F) and transmission electron microscopy (TEM, JEOL, JEM-3010). The phases of the obtained products are collected on a Bruker D8 Advance X-ray diffraction (Cu K α radiation, λ = 0.15406 Å in a 2θ range from 10° to 80° at room temperature). Raman experiments are performed using a DXR spectrometer with the 532 nm excitation line. UV-visible diffuse reflectance spectra are recorded within the 200-800 nm wavelength range using a Shimadzu UV2450 spectrometer. Fourier transform infrared (FT-IR) spectra are recorded on a Thermo Nicolet NEXUS-670 spectrometer with KBr pellets in the 4000-400 cm^-1^ region.

**2. Photocatalytic activity test**

The performance of the photocatalytic materials was detected by the optical system for the photocatalytic reaction which was composed of a 350 W Xe lamp and a cut off filter (λ> 420 nm). The original solution was prepared by adding 100 mL dyes (MO-10ppm, RhB-10ppm, Phenol-60ppm), and 50 mg composites into the solution. The solution was ultrasonicated for 30 min to form the aqueous dispersion, and then transferred into a sealed glass beaker to stir in the dark for 30 min to ensure absorption-desorption equilibrium. After visible light illumination, 4 mL of samples were taken out at regular time intervals (20 min) and separated through centrifugation (10000 rpm, 10 min). UV-vis absorption spectra are recorded at different intervals to monitor the reaction using by using a Lambda 25 UV/vis spectrophotometer. The photocatalytic degradation efﬁciency (E) of dyes was obtained by the following formula:

E= (1-C/C_0_)* 100 %= (1-A/A_0_)*100%

Where C was the concentration of the RhB solution at reaction time t, C_0_ was the adsorption/desorption equilibrium concentration of RhB (at reaction time 0); A and A_0_ were the corresponding values.

**3. Bactericidal activities of GCD**

The bacterial liquid was standardized (1*10^5^ -1*10^7^ CFU/mL) by measuring the absorbance using an optical density (OD) (A 625=0.1; SP-1800 Spectrophotometer, Pye-Unicam, Cambridge, UK.). For the examination of flat colony counting method, 200 ppm GCD was dispersed in the sterilized PBS solution, followed by the addition of 10^6^ CFU/mL bacteria for further incubation in a temperature-controlled rotary shaker at 20 °C for 0, 24 h, 48 h, respectively.

For the characterizations of bacteria before/after the treatment of GCD, 10 μL of each specimen was loaded on TEM copper grids followed by staining with tungstophosphoric acid. After air-dry the copper grids, the samples were examined using the TEM (JEOL JEM-2100) as described above.


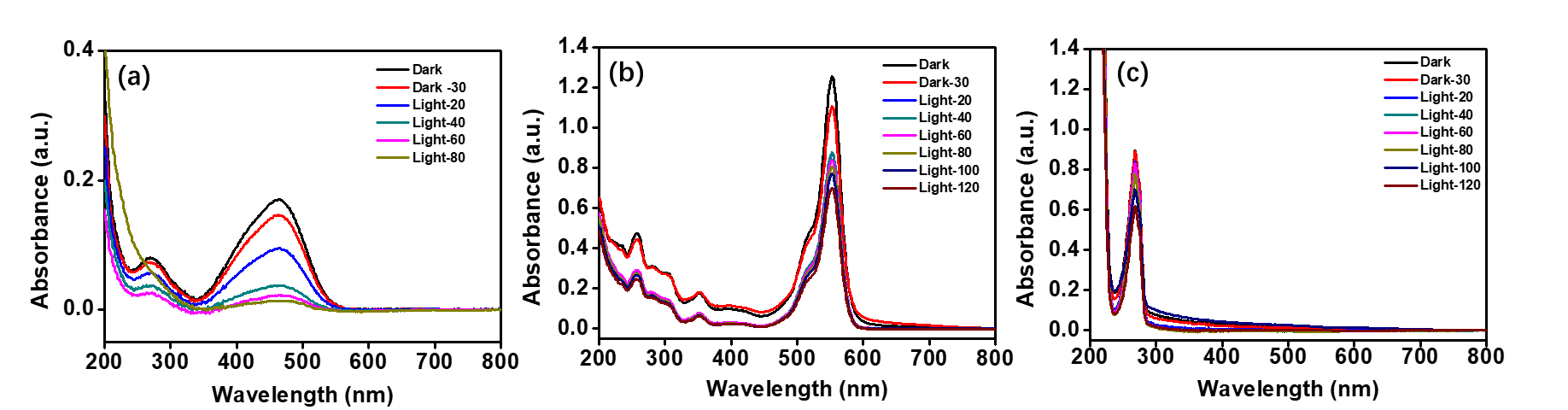


Figure S1. UV-vis absorption spectra of GCD for MO (a), RhB (b) and phenol (c).

1. [↑](#footnote-ref-1)
2. [↑](#footnote-ref-2)
3. [↑](#footnote-ref-3)
